# Novel approaches to risk stratification to support malaria elimination: an example from Cambodia

**DOI:** 10.1186/1475-2875-13-371

**Published:** 2014-09-19

**Authors:** Jonathan Cox, Siv Sovannaroth, Lek Dy Soley, Pengby Ngor, Steven Mellor, Arantxa Roca-Feltrer

**Affiliations:** Department of Disease Control, Faculty of Infectious and Tropical Diseases, London School of Hygiene and Tropical Medicine, London, UK; Malaria Consortium, Phnom Penh, Cambodia; National Center for Parasitology, Entomology and Malaria Control, Phnom Penh, Cambodia; National Institute of Public Health, Phnom Penh, Cambodia

**Keywords:** Malaria, Cambodia, Surveillance, Stratification, Elimination

## Abstract

**Background:**

Accurate malaria stratification is essential for effective targeting of interventions but represents a particular challenge in pre-elimination settings. In these settings transmission is typically sufficiently low and spatially heterogeneous to warrant a need for estimates of malaria risk at sub-district or village level but is also likely to be sufficiently high to render the type of decision support systems appropriate to the final stages of malaria elimination impractical. In such a scenario it is arguably more feasible to strengthen existing passive malaria surveillance systems so that routinely generated case data can provide an effective basis for stratifying malaria risk. This paper explores the utility of routine malaria surveillance data for the stratification of malaria risk in Cambodia, where the target is malaria elimination by 2025.

**Methods:**

A malaria information system (MIS) was developed to generate timely, routine data on temporal and spatial variations in malaria cases reported through public health facilities and village malaria workers (VMWs). The MIS was implemented across all malaria endemic districts in the country during 2010–11. In 2012 MIS data were extracted and assessed on the basis of coverage and completeness. Village-level incidence estimates for 2011 were generated using predefined data inclusion criteria.

**Results:**

In 2011, the MIS covered 681 health facilities and 1,489 VMW villages; the overall completeness of monthly reporting was 82& and 97& for health facilities and VMWs respectively. Using these data it was possible to estimate malaria incidence for 89& of villages covered by the MIS. The resulting stratification highlights the highly heterogeneous nature of malaria transmission in Cambodia and underlines the importance of village-level data for effective targeting of interventions, including VMWs. Challenges associated with implementing the MIS and the implications of these for developing viable and sustainable MIS in Cambodia and elsewhere are discussed.

**Conclusions:**

This study demonstrates the operational feasibility of introducing a system to routinely generate village level malaria case data in Cambodia. Although resulting incidence estimates are subject to various limitations and biases the data provide an objective, repeatable basis for a dynamic system of stratification which is appropriate for guiding the transition between malaria pre-elimination and elimination phases.

**Electronic supplementary material:**

The online version of this article (doi:10.1186/1475-2875-13-371) contains supplementary material, which is available to authorized users.

## Background

Malaria stratification involves the classification of geographical areas or localities according to the risk of malaria and has long been recognized as an essential element of efficient resource allocation and a prerequisite for the rational targeting of interventions. Efforts to use malaria stratification to directly guide control and prevention activities date back to the 1940s [1]. Although a wide variety of stratification methodologies and typologies have been used since, most have involved supplementing existing malariometric data with information on the spatial distribution of key determinants of transmission risk including climate, ecology, geomorphology and the presence or abundance of key vector species [2, 3]. In essence this basic framework has not changed substantially over time, although developments in geographical information systems (GIS), remote sensing and geostatistical techniques have led to major advances in the sophistication and reproducibility of spatial risk estimates [4, 5].

Operational stratification at the national level has typically involved mapping out areas that share common epidemiological characteristics in terms of transmission rates, seasonality and vector and parasite species. From a spatial perspective, data for malaria and other variables included in the stratification are often somewhat generalized, which means that stratification products derived from these data are unable to capture fine scale geographical heterogeneity in malaria risk. Within a typical malaria control scenario, stratification efforts are usually focused on province or, in some settings, district level outcomes in line with the principal unit of planning and implementation of interventions. However, in moderate and low transmission settings characterized by marked spatial heterogeneity of malaria risk, stratification products need to more spatially specific in order to facilitate sufficiently precise targeting of interventions, ideally at sub-district or village level [6–8]. In an elimination phase the development of specific spatial decision support systems may be justified [9], but in the early stages of pre-elimination there is also an argument for strengthening and/or modifying malaria surveillance systems so that routinely generated case data can provide an effective basis for risk stratification and guide the transition from pre-elimination to elimination phases [10–12].

Cambodia is an example of a country that, having achieved substantial reductions in malaria morbidity and mortality in the last decade, is now making the shift from a control phase to an elimination phase. The Cambodian National Strategic Plan for Elimination of Malaria aims to ensure that no artemisinin resistant malaria parasites are detected in Cambodia by 2015, there are no malaria deaths and *Plasmodium falciparum* malaria is eliminated by 2020 and *Plasmodium vivax* and other forms of malaria are eliminated by 2025 [13]. Within this context the National Centre for Parasitology, Entomology and Malaria Control in Cambodia (previously the Centre National de Malariologie; CNM) has been exploring a range of options for strengthening malaria surveillance and optimizing the targeting of malaria interventions through a more refined malaria stratification risk approach [14]. A core element of this activity has been the development of a Malaria Information System (MIS) to provide timely data on temporal and spatial variations in malaria case numbers at village level across all malaria endemic districts in the country. This paper describes the process of developing the MIS and explores the potential utility of the platform for malaria risk stratification. MIS coverage and reporting completeness are assessed, derivative estimates of village incidence data are described and the strengths and limitations these estimates discussed. The paper also explores challenges associated with the development and implementation of the MIS and addresses the wider applicability of this approach to other pre-elimination and elimination settings.

### Malaria surveillance and stratification in Cambodia

Over recent years there has been a marked decline in the number of treated malaria cases reported by government health facilities in Cambodia - from approximately 130,000 cases in 2000 to around 45,000 in 2012 (CNM, unpublished data). Over the same period the percentage of infections represented by *P. falciparum* has fallen from over 90& to less than 60&. However, these national statistics mask substantial heterogeneity in malaria risk between geographic areas and different population groups. In particular, living or working in forested areas has long been recognized as the primary risk factor for malaria infection in Cambodia and other Mekong countries where *Anopheles dirus* and *Anopheles minimus* represent important vectors [15, 16]. This is manifested by marked variations in malaria prevalence over relatively short distances (*e.g*. [17, 18]).

In Cambodia, the importance of using information on the spatial heterogeneity of malaria risk to guide malaria control activities has long been recognized. For over a decade CNM has used a system through which individual villages are allocated to defined malaria risk strata that in turn inform explicitly the deployment of malaria interventions including long-lasting insecticide treated nets and village malaria workers (VMWs) who provide early detection and treatment. To date this stratification has been primarily ecological, with villages being assigned a malaria risk category based on their distance to forest. This process began in 2001 and involved overlaying village locations and remotely-sensed forest distribution data in a GIS. However, total primary forest cover in Cambodia has declined by around a third in the last decade [19] and as available forest maps have become progressively outdated, CNM has opted to periodically update village risk categories using an informal system of national and local expert opinion on changing ecological risk (S. Sovannaroth, CNM, personal communication). The main weaknesses of this approach are (a) that stratification is based on a proxy of malaria risk rather than a more direct measure; and (b) that the process of classifying village-level risk is inherently subjective and thereby effectively non-reproducible.

Since 2004 routine surveillance of malaria cases has been carried out through the general framework of the Cambodia Health Management Information System (HMIS). The HMIS faces a number of operational constraints [20] but from the perspective of malaria surveillance its principal limitations relate to the absence of specific data on parasite species and the fact that health related information is gathered only down to the level of the health facility. Most significantly the HMIS includes only data for cases presenting to government health facilities, as there is not a mechanism for districts to include data reported by VMWs. Since its introduction in 2001, the VMW network in Cambodia has been substantially scaled up [21, 22] and today covers more than 1,500 villages in 18 provinces (unpublished CNM data). It has, therefore, fallen to CNM to manually integrate HMIS and VMW data to derive comprehensive estimates of case numbers at the national level.

## Methods

### Development of the MIS

**Figure 1:**
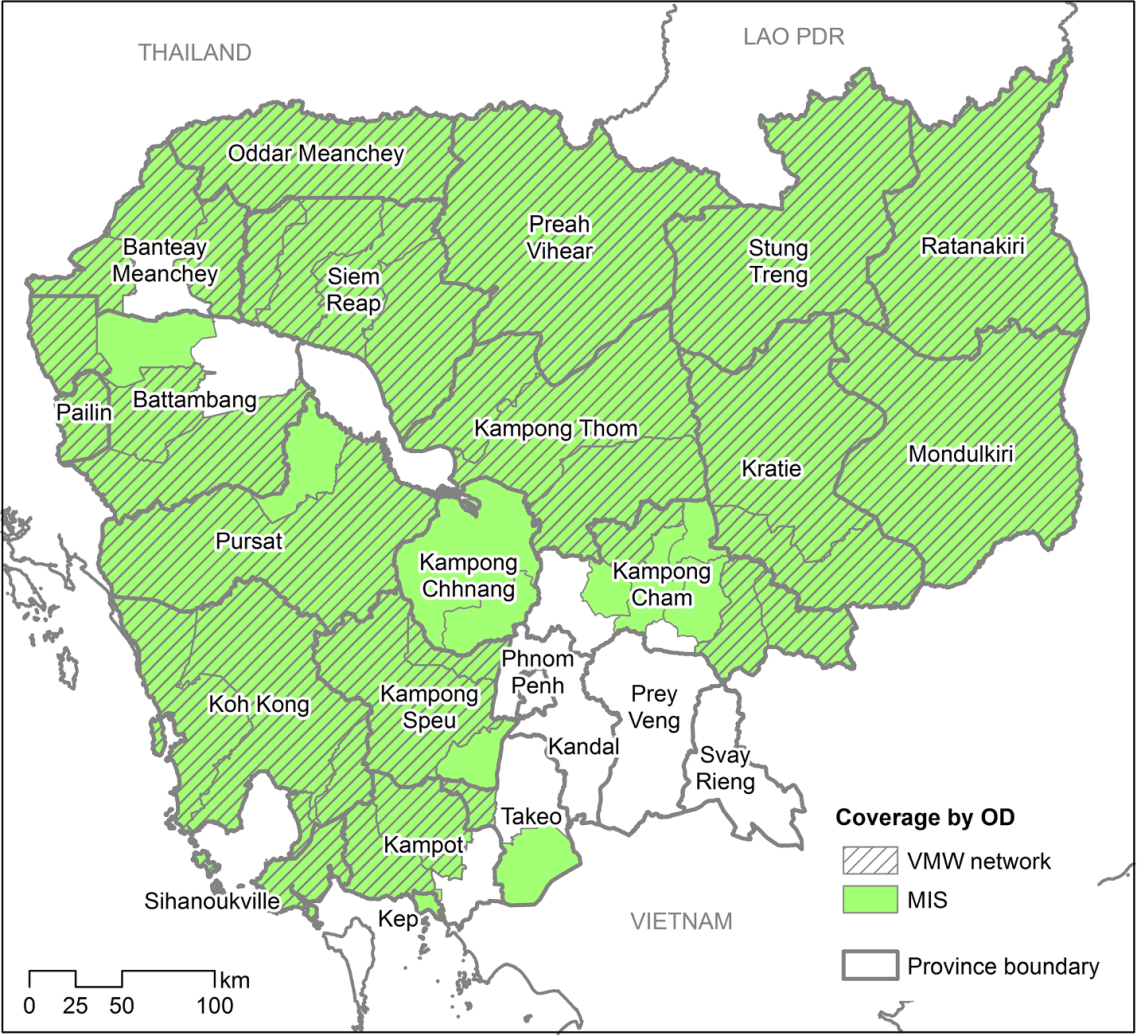
**Map of operational districts in Cambodia covered by the MIS and VMW network in 2011.**

In late 2008, Cambodia and Thailand embarked on a joint strategy for the containment of artemisinin-tolerant malaria parasites in South-East Asia (ARCE), which involved the introduction or scaling up of a number of interventions in key districts on both sides of the Thai-Cambodia border [23]. Within Cambodia a key element of the ARCE strategy has been the strengthening of systems for malaria surveillance and stratification. This has included the development of a MIS as a platform for village level case reporting and incidence-based stratification [24]. With technical assistance from Malaria Consortium (MC), development of the MIS within CNM began in 2009, with activities initially focusing on four provinces in western Cambodia. In 2010 the database was scaled up to cover all operational (health) districts (OD) included within CNM’s malaria control strategy. ODs are amalgamations of administrative districts and each typically covers a population of 100,000-200,000. Of a total of 78 ODs in Cambodia, 45 are considered to be at risk of malaria and are hence included within the MIS (Figure 1). The remainder, located mainly of the south-central part of the country (including the capital, Phnom Penh) are considered free of malaria transmission.

The MIS contains a variety of data relating to malaria case numbers and intervention coverage [14]. In order to facilitate routine monthly reporting of data for individual malaria cases a new (paper) form was introduced to capture information from existing health facility registers. As existing monthly VMW reports already included a line listing of individuals diagnosed for malaria, new forms were not required for VMWs. Each month health facilities routinely send copies of paper forms (containing data for individual cases presenting at the health facility or to VMWs within the health facility catchment) to the relevant OD office. Staff at the OD office then enter data from the paper forms into a customized Access database (Microsoft Corporation, Redmond, USA). Each patient record includes information on residence, which for purposes of consistency is selected from a standardized list of village names and associated unique identifier codes. Monthly updated data extracts are sent to CNM by email, where they are automatically applied to the national database. The database incorporates a range of queries that can be used for data exploration, summarization and reporting [14].

The MIS database platform was developed within CNM with technical assistance from MC over a period of three months. Subsequent roll-out of the database to individual ODs took 12 months to complete and in each case was preceded by training from a surveillance and data manager specialist (CNM and MC staff respectively). OD staff were asked to collate malaria data retrospectively to the beginning of 2010 where feasible. The assessment of MIS data reported in this paper was carried out in December 2012 and incorporated data collected by the system within the period January 2010 to June 2012. For this assessment data management and analysis were carried out in Stata (version 12; StataCorp, College Station, USA) and ArcGIS (version 10.1; ESRI, Redlands, USA). In practice this involved developing individual datasets comprising line listings of all malaria cases reported by health facilities or VMWs, together with linked datasets on the monthly reporting status of individual facilities and VMWs. Initial data quality screening of case data was carried out to identify and remove records that lacked either a correct village code or a definitive diagnosis. The cleaned case data were aggregated by village and month and then merged with tables containing data on village attributes (*e.g*. existing CNM risk category) and monthly reporting by the relevant supervising health facility.

## Results

### MIS coverage and completeness

#### Health facility data

As noted above, coverage of the MIS is restricted to 45 ODs targeted for malaria control activities by CNM (Figure 1). The MIS covers 681 health facilities (61& of the national total), the majority of which constitute health centres (n = 569). The remainder constitutes a mixture of district/provincial hospitals (n = 48) and former district hospitals (FDH; n = 65). A total of 633 health facilities (91.5&) reported data at some point in the reporting period January 2010 to June 2012. Taken together, 93.3& of health centres and FDH reported at some point in this period, while in contrast the corresponding figure for district and provincial hospitals was 66.7&. As all ODs within the MIS were asked to collate village-level malaria cases retrospectively back to the start of 2010, in principle all health facilities should have submitted 30 monthly reports up to June 2012. In reality the overall reporting rate across all MIS health facilities (expressed as the percentage of monthly reports successfully submitted) was 76.1&. Reporting rates were substantially higher in 2011 (at 81.6&) than in either 2010 or 2012 (70.2& and 77.0&, respectively). The relatively low reporting rate in 2010 suggests that ODs experienced difficulties in entering case data retrospectively. Effective “prospective” reporting rates (calculated for each facility from the point at which data were first reported) were considerably higher, at 93.9&.

#### VMW data

The MIS covers all ODs currently included in the VMW network (Figure 1). Within this network VMWs are deployed in selected villages within the catchments of targeted health facilities. VMWs carry out rapid diagnostic tests on individuals suspected of having malaria and treat or refer test-positive cases. VMWs submit data to their supervising health centre on a monthly basis. At the start of 2012 just under a quarter (24.5&) of health facilities in the MIS supervised VMWs and 15.1& of villages within the MIS were included in the VMW network. The network is dynamic in the sense that the number of active VMWs in any given OD may increase over time as the network expands or, less commonly, decrease over time as VMWs are withdrawn. In order to determine overall VMW reporting completeness in the current exercise a VMW was assumed to be effectively “active” throughout the period spanning their first and last reports. Based on this assumption the overall VMW reporting rate over the study period was 96.5&. The reporting rate was lowest in 2010 (95.3&) and highest in 2012 (97.7&). The number of VMWs considered active in 2010, 2011 and the first half of 2012 was 1385, 1489 and 1520 respectively.

### Developing an incidence-based stratification

Given the relatively incomplete state of the 2010 health facility data, only data for 2011 were used for the development of the stratification product. As described above, case data from both health facilities and VMWs were available as line listings of all positive malaria cases. In 2011, a total of 86,684 malaria cases were reported, of which 48,497 (55.9&) had presented to VMWs. Overall only a small number of cases (1,135, or 1.3& of total reported cases) had to be excluded from subsequent analysis because they lacked either an appropriate village code or a definitive malaria diagnosis. In total, 5,713 villages reported malaria cases in 2011, which represents 57.8& of all villages within the MIS zone. Of 1,489 VMW villages included in the MIS in 2011, 1,371 (92.1&) reported malaria cases in 2011.

#### Inclusion/exclusion criteria for stratification

**Figure 2:**
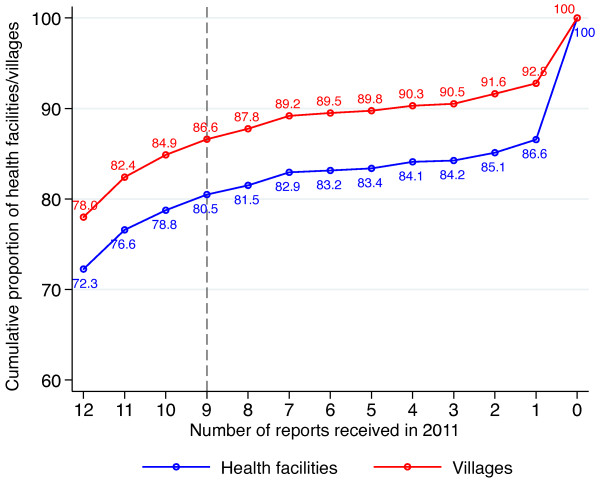
**Cumulative proportion of health facilities and associated villages submitting between 0 and 12 monthly reports in 2011.** The vertical dashed line indicates the reporting threshold (9 months of reporting) selected in the current study.

As noted above, the reporting completeness from health facilities in 2011 was 81.6&, while the corresponding figure for VMWs was 97.0&. Therefore, although the majority VMWs and health facilities were able to achieve full reporting in 2011, a considerable number failed to report in some or all months. In terms of stratification, incomplete reporting will have an impact on the accuracy of incidence estimates and it is, therefore, necessary to develop suitable inclusion/exclusion criteria to maximize the coverage and reliability of the resulting stratification. Defining these criteria essentially involves achieving a trade-off between (a) minimizing the number of gaps in the spatial coverage of the final product and (b) maximizing the accuracy of incidence estimates for individual villages. In practice this comes down to deciding on a minimum number of reporting months in a given year that can be said to provide an adequate, representative estimate of malaria incidence. In the case of health facility data in 2011 this trade-off is illustrated in Figure 2, which indicates how the proportion of health facilities (and associated villages) that can be included in the stratification varies depending on which threshold for “completeness” is applied. At one extreme, applying a very strict decision-rule under which only data from facilities with complete reporting (*i.e.* 12 reports in 2011) are included would mean that 72.3& of facilities (or 78.0& of villages) could be included in the stratification. Making this rule less strict, for example to include facilities that reported data in nine or more months, would increase the proportion of facilities and villages that could be included in the stratification to 80.5& and 86.6& respectively. This fairly substantial increase in coverage would arguably be achieved with relatively little risk to the quality of resultant village-level incidence estimates.

In reality the choice of threshold is somewhat arbitrary, although this type of cumulative proportion plot may act as a useful guide. Depending on the seasonal pattern of transmission it may also be appropriate to develop secondary inclusion/exclusion criteria that relate to key months of the year. In the current exercise seasonality was assessed by deriving metrics previously proposed by Roca-Feltrer *et al.* [25] using a six-year time series of malaria data from the Cambodia HMIS (see Additional file 1). Results of this analysis suggested that malaria seasonality in Cambodia is not especially pronounced, although it is worth noting that more than 70& of cases occur between June and December.

In the current exercise, data inclusion criteria were chosen in consultation with CNM and partners. A threshold of nine monthly reports over the year, including at least three reports from the high transmission season (June to December), was selected. For each village covered by the MIS this threshold was applied to both health facility and VMW reports (where applicable). Incidence estimates for a village were deemed to be valid if reporting from either source met this threshold. In total, 1,048 villages were excluded from the stratification on this basis, representing 10.6& of all villages within the MIS. Just over half of the excluded villages had submitted no reports in 2011; the remainder had submitted an insufficient number of reports to meet the stratification inclusion criteria. The proportion of villages qualifying for the stratification varied by OD, but with no obvious subnational pattern (see Additional file 2, Figure 1).

#### An incidence-based stratification for 2011

Village-level case data were matched to population data available from existing census records (2008) or, where available, to more recent village population data collected as part of mass distributions of long-lasting insecticidal nets. Population estimates were not available for 441 (4.5&) of MIS villages. In total, 408 villages that would otherwise have qualified on the basis of reporting completeness were excluded from the stratification, leaving a total of 8,420 villages – or 85.3& of all villages covered by the MIS – within the stratification. The overall number of villages falling within specified malaria incidence categories is presented in Table 1. Overall 36.2& of MIS villages reported no malaria in 2011 and a majority (65.4&) reported fewer than five cases per thousand population. Within the MIS zone median village-level malaria incidence per OD ranged from 0 to 33.4 per 1,000 population in 2011 (overall median village incidence = 1.9), with the highest rates being in eastern and northern parts of the country (Ratanakiri, Stung Treng, Mondulkiri, Preah Vihear and Kratie), as well as in Koh Kong in the west (Additional file 2, Figure 2). Overall, *P. falciparum* and mixed infections accounted for 57.7& of treated cases. However, this proportion varied considerably by OD (range 18.6-88.4&), with *P. vivax* predominating in some low malaria transmission areas of western Cambodia (Banteay Meanchey, Oddar Meanchey and Pailin).

**Table 1:** **Numbers of villages and population size for defined categories of malaria incidence within the MIS zone**

	All malaria species				*P. falciparum*and mixed infections*				*P. vivax*			
Annual incidence rate per 1,000 population	Number of villages (& of total)		Population (1000s) (& of total)		Number of villages (& of total)		Population (1000s) (& of total)		Number of villages (& of total)		Population (1000s) (& of total)	
0	3052	(36.2)	2225	(30.6)	3860	(45.8)	2921	(40.2)	4521	(53.7)	3499	(48.2)
>0-1	328	(3.9)	720	(9.9)	413	(4.9)	908	(12.5)	351	(4.2)	766	(10.5)
>1-5	2126	(25.2)	2083	(28.7)	2024	(24.0)	1900	(26.1)	1782	(21.2)	1712	(23.6)
>5-20	1612	(19.1)	1373	(18.9)	1314	(15.6)	1049	(14.4)	1030	(12.2)	875	(12.0)
>20-50	623	(7.4)	506	(7.0)	381	(4.5)	284	(3.9)	412	(4.9)	275	(3.8)
>50	679	(8.1)	359	(4.9)	428	(5.1)	203	(2.8)	324	(3.8)	139	(1.9)

*P. falciparum and mixed infections are combined because results from Pf/PAN combo rapid diagnostic tests (used by the VMWs in 2011) showing two positive lines in addition to the control line, were systematically classified by CNM as *P. falciparum.*

As noted previously, Cambodia currently has a stratification system within which all villages in target ODs are allocated a risk status based principally on distance to forest: 0–500 m (risk category 1), 500–1,000 m (category 2), 1,000-2,000 m (category 3). Villages situated more than 2,000 m from forest are not considered to be at risk of transmission and are therefore not targeted for malaria control interventions. To gauge the effectiveness of the current system we estimated malaria incidence MIS villages according to their CNM risk category. Incidence data from each village were expanded to simulate individual data, ensuring that estimates had the correct rate and corresponding standard error. Poisson regression analyses were used to compare incidence rates across risk categories allowing for the clustering of data within each village. Results indicated a clear trend of increasing malaria incidence associated with CNM risk category (risk category 1: 42.93 per 1000 (95& CI 42.53-43.34); category 2: 16.78 (16.54-17.03); category 3: 7.89 (7.71-8.00); “no-risk” villages: 2.36 (2.32-2.42); likelihood ratio test for linear trend: p < 0.001). The fact that the CNM system is able to differentiate so effectively between different levels of malaria incidence is perhaps surprising, but is likely to reflect the fact that over time the existing CNM system has been refined to take on more nuanced perceptions of local transmission risk, which incorporates local knowledge on case patterns as well as ecology (S. Sovannaroth, CNM, personal communication).

#### Targeting of surveillance activities

As noted previously, the MIS currently covers 45 of 78 ODs in Cambodia. These represent target ODs for malaria control activities, with the assumption that ODs outside this zone (see Figure 1) do not support malaria transmission. To assess the appropriateness of this targeting of malaria surveillance, routine data from the Cambodia HMIS were used to determine the number of malaria cases reporting in ODs not included in the MIS. In 2011 the total number of confirmed malaria cases reported outside the MIS zone was 5,250 – or 5.0& of all confirmed cases reported nationally in 2011. Of these, more than half presented at national referral facilities in Phnom Penh. A relatively small number of cases (1,728, or 1.6& of all reported cases nationally) presented in the 16 ODs that are geographically contiguous with the MIS zone and which, realistically can, therefore, be considered potential instances of local transmission outside the MIS zone.

## Discussion

This paper describes the process of developing an incidence-based malaria stratification at village level using malaria case data generated routinely through a MIS. The MIS has been developed by CNM as a means of generating malaria data that are both more timely and more spatially specific than data available through the existing national HMIS. As a parallel reporting system the MIS does introduce new tasks at health facility and OD level, although in designing and developing the MIS the intention was to keep the burden associated with these activities as small as possible by minimizing the number of variables to be reported and by automating a number of core data manipulation, analysis and reporting tasks (this also applies to data management at the national level, where CNM uses the MIS to generate regular national malaria bulletins [14]). To date no qualitative evaluation has been carried out to document perceptions of MIS “users” at the peripheral level; however, the relatively high reporting rates described in this paper suggest that the MIS is both operationally viable and, with appropriate support, sustainable as a routine data collection system. In this case support came in the form of monetary incentives to health facility and OD MIS “users” of US$50 and US$90 a month respectively since the start of the MIS roll out in 2010. However, these incentives were discontinued in 2012 due to changes in donor/funding policies changes and the lower reporting rates observed in 2012 may reflect this change. This indicates that the system requires maintenance and incentives to ensure quality and completeness of data. A joint CNM-Malaria Consortium assessment of the MIS, scheduled for late 2014 will assess the impact of variations in monetary incentives on surveillance activities at various levels in the health system including VMWs, health centre and OD staff. The assessment will be used to guide future developments of the MIS as Cambodia continues to develop strategies to support malaria elimination.

Although OD staff were not always able to collate retrospective health facility data, the overall reporting rate for prospective data was 93.9& (the corresponding rate for VMW data was 96.5&). However, a substantial number of health facilities failed to report data at any point in 2011 (of the 1,048 villages excluded from the stratification just over half submitted no reports) and OD-level reporting rates were also highly variable. In addition, reporting rates for health facilities did drop off slightly at the beginning of 2012 and the reasons behind this need to be fully explored. Moving forward it is important that mechanisms are introduced that allow CNM and district staff to continuously monitor reporting rates and to facilitate effective follow up with individual ODs and/or health facilities when necessary. It is relatively easy to identify health facilities that fail to report in a timely manner using basic data queries within the MIS; however standard procedures are also needed to ensure that this information is acted upon.

As well as operational challenges associated with maintaining and sustaining the MIS it is also important to recognize that the initial development of the system was not a trivial exercise. The roll out period of the Cambodia MIS took approximately one year and required intense engagement from the central level to train VMWs and government staff (particularly at the OD level) on the new reporting forms, data entry and reporting procedures. On-the-job and refresher trainings at OD levels during that period was required and probably contributed to the relatively low reporting rates observed in 2010.

Geographically, the coverage of the MIS is restricted to 45 ODs targeted for malaria control activities by CNM. Analysis of national HMIS data for 2011 indicated that a small proportion (5.0&) of malaria cases were diagnosed at health facilities outside this MIS zone. The majority (67.1&) of these were reported in ODs that do not border endemic ODs and it is reasonable to assume that these cases were either imported or are the result of internal migration within Cambodia. However, around a third of the HMIS malaria cases not captured by the MIS were reported by ODs bordering endemic areas and these cases may well indicate pockets of local transmission. A systematic analysis of HMIS data at the level of health facility catchment is therefore required in order to inform potential scaling up of the MIS to additional ODs. Linked to this point it should be noted that even within the MIS system there is likely to be a substantial (but unknown) number of infections that are attributed to the wrong location. In Cambodia, where travel is a major risk factor for malaria, infection may commonly occur away from a patient’s village of residence. Although VMWs do record whether malaria cases are considered residents or migrants for a given village, there is currently no way to efficiently capture specific information about travel history in a routine setting. In practice this could mean that two localities that have comparable levels of malaria incidence on paper in reality experience quite different levels of transmission and it is therefore important that any incidence-based product is reviewed in the light of epidemiological risk factors that determine local transmission risk. As a first step to address this issue, VMW data should be routinely analyzed to assess the proportion of malaria cases seen in a given village that are classified as “migrants”. That information could support interpretation of findings and subsequent categorizations of local versus imported malaria cases. Over the longer term it would be beneficial to introduce a standardized, clearly documented system of case classification (for use by VMWs and health facility staff) so that local and imported cases can be more effectively distinguished.

Given that the purpose of a malaria stratification is not simply to map cases but to guide appropriate intervention strategies, it is also important that key elements of malaria epidemiology (*e.g*. vector ecology, socio-economic characteristics of the at-risk population) are taken into account when determining locally appropriate mixes of interventions or the appropriateness of implementing reactive case detection on the basis of passively detected cases [2, 26, 27]. In Cambodia the intention is that expert panels at national, province and OD level will screen village-level classifications based on the MIS estimates of incidence and upgrade or downgrade the risk category of specific villages where this is deemed to be appropriate. This system has some similarities with “micro-stratification” exercises that have recently been conducted elsewhere in South East Asia [28–30]. The advantage of the MIS approach is that the core malariometric data required for the micro-stratification (*i.e.* village-specific incidence data) are generated automatically, rather than as an inherent (and time-consuming) part of the local assessment exercise allowing for regular updates that can guide the transition between the various phases of malaria control, pre-elimination and elimination.

Evidence presented in this paper demonstrates that nationally reported cases data are not always “fanciful”, as has been maintained elsewhere [31], although it should be recognized that no routine surveillance system can be considered to be comprehensive. In Cambodia, where some private providers are authorized to test and treat cases of uncomplicated malaria, a significant percentage of patients will initially seek treatment outside the public health system [32]. Linked to the MIS, a system for collecting data from private clinics and pharmacies is currently being piloted in ten ODs. A system that allows military and police to report malaria cases through the MIS has also recently been implemented. Even with these initiatives it will not be possible for the MIS to capture all malaria cases; accordingly village-level incidence data should be seen as a guide to local transmission intensity and as a means of representing relative differences in malaria risk between localities, rather than as definitive estimates of malaria case numbers. Data from these multisectorial sources could, however, be used to triangulate malaria trends based on data derived from public health systems.

It should also be recognized that within the public health system gaps in case reporting can be considered the norm and will always have an impact on the quality and completeness of village-level stratifications. In the current exercise village-level incidence estimates for 2011 were deemed to be valid if at least nine health facility or VMW reports had been received over the course of the year, including at least three reports from the high transmission season. In practice incidence estimates could be generated for 90& of the 9,765 settlements located in the MIS zone. Of those villages for which incidence could not be estimated, more than a quarter (28&) were missing population (denominator) data, rather than malaria case (numerator) data and these data gaps are relatively easy to rectify. Gaps in the stratification owing to missing malaria data are less easy to solve. In the short term CNM intends to use its existing system of expert opinion to allocate risk categories to villages excluded from the incidence-based stratification. In the medium term it may be feasible to incorporate geostatistical techniques as a means of imputing these missing data (see, for example, [5]) and also as a means of semi-automated mapping [33]. For most control programmes it is likely that substantial strengthening, particularly in terms of staff time, capacity and skills, would be required to support this type of application.

Data presented in this paper demonstrate a highly heterogeneous distribution of malaria cases between individual villages. Taking only the MIS villages into account over half (51.6&) of all malaria cases originated from 5& of villages, while 85.9& of cases came from 20& of villages. The MIS is an effective way of flagging these high-risk villages and for gauging the degree to which interventions are being appropriately targeted. One very notable result from the current analysis is that 8& of villages within the VMW network did not report any cases of malaria in 2011 (in Battambang and Pailin the proportion was 16& and 17& respectively). Village-level incidence data from the MIS can help guide the deployment of VMWs and ensure that the scale and geographical focus network are appropriate and that the network is sufficiently dynamic to adapt to a rapidly changing epidemiological context. VMWs can either be redeployed to higher risk villages or, alternatively, the responsibilities of VMWs in villages with very low case numbers can be redefined, for example to facilitate active case finding. In this way the MIS can inform not only choices of intervention but also identify geographical areas where it might be appropriate to switch from passive to active case detection. It also provides a means of targeting areas for the introduction of spatial decision support systems or other mHealth solutions that are predicated on identifying and responding to individual incident cases.

There are limitations to the current assessment of the MIS that require further consideration and analysis. Firstly, it should be noted that the assessment of reporting completeness was limited to the submission of monthly reports and did not incorporate an assessment of the accuracy and completeness of the data contained in those reports. A separate data quality audit based on field validation would provide important additional insights. Secondly, there was no access to data relating to the timeliness of data reporting (*i.e.* the median reporting lag between diagnosis and receipt by CNM). An important element of the rationale for introducing the MIS was to speed up the monthly reporting process. In theory, the MIS should be able to achieve by replacing the pre-existing system of sending paper reports via Provincial Health Departments with a system through which ODs send data directly to CNM by email. However, it was not possible to gauge the effect of the MIS on timeliness as part of this assessment.

## Conclusions

This study demonstrates the operational feasibility of introducing a system to routinely generate village level malaria case data in Cambodia, although the practical challenges associated with implementing and sustaining such as system should not be underestimated. Data from the MIS demonstrate a highly heterogeneous distribution of malaria cases between individual villages in Cambodia as well as between districts. Although village-level incidence estimates are subject to various limitations and biases the data provide an objective, repeatable basis for a dynamic system of stratification which is appropriate for guiding the transition between malaria pre-elimination and elimination phases. In Cambodia it is likely that the MIS will be particularly useful for guiding the deployment of VMWs and as a basis for phasing in new surveillance activities predicated on active case finding.

## Electronic supplementary material

Additional file 1: **Defining malaria seasonality in Cambodia.** This file describes an assessment of the degree of seasonality in malaria data for Cambodia. This information was used to inform data inclusion criteria for the malaria stratification process. (PDF 333 KB)

Additional file 2: **Maps of key district-level outcomes.** This file includes maps that illustrate the spatial distribution of three outcomes of the analysis down to the level of the operational district: proportion of villages contributing to the 2011 stratification; malaria incidence in 2011; *P. falciparum* as a proportion of all malaria cases. (PDF 3 MB)

## Abbreviations

ARCE: Containment of artemisinin-tolerant malaria parasites in South-East Asia
CNM: National Center for Parasitology, Entomology and Malaria Control
FDH: Former district hospital
GIS: Geographical Information System
HMIS: Health Management Information System
MC: Malaria Consortium
MIS: Malaria Information System
OD: Operational district
VMW: Village malaria worker.
