# Supplementary material for: Novel approaches to risk stratification to support malaria elimination: an example from Cambodia
Source: Malar J. 2014 Sep 19;13:371. doi: 10.1186/1475-2875-13-371 (PMC4177243; doi:10.1186/1475-2875-13-371)
Supplement: Supplementary file 1 — Additional file 1: Defining malaria seasonality in Cambodia. This file describes an assessment of the degree of seasonality in malaria data for Cambodia. This information was used to inform data inclusion criteria for the malaria stratification process. (PDF 333 KB) [file 12936_2014_3402_MOESM1_ESM.pdf]

Additional file 1
Defining malaria seasonality in Cambodia

In the absence of a universally recognized, standard approach for describing malaria seasonality we adopted the method proposed by Roca-Feltrer *et al*. [1]. This method is simple to implement and has been shown to discriminate well between sites based on different degrees of seasonality.

Analysis was carried out using a 6-year monthly time series (2006-2011) for all malaria cases reported through the Cambodia Health Management Information System (HMIS). Seasonality was determined separately for cases diagnosed with *Plasmodium falciparum* and *P. vivax*, as well as for all treated cases. Prior to 2009 the RDTs used for routine diagnosis in Cambodia were only able to diagnose *P. falciparum* infections ("combo" tests, which allow diagnosis of *P. vivax* were introduced in 2009). For this reason only data for the period 2010-2011 were used to describe seasonality of *P. vivax* cases.

The cumulative percentage was calculated for each type of episode for each consecutive 6-month period in turn, using a rolling starting month. The 6-month window with the highest cumulative percentage was defined as the "concentrated period". The results of this analysis are shown in Figure 1, which indicates that for all episode types the concentrated period started in July. The cumulative percentage of episodes represented by the concentrated period was relatively constant across the different indicators (61.3% for treated cases, 63.4% for *P. falciparum* cases and 61.6 for *P. vivax* cases). Therefore none of these indicators can be seen as demonstrating "marked" seasonality (defined in [1] as 75% or more of cases occuring in =<6 months), or even "border-line marked seasonality" (75% or more of cases occuring in 7 months). It is, however noticeable that for each of the indicators the number of episodes was lowest in the period February-May. Moreover, in all cases the 7-month period from June to December accounted for more than 70% of total episodes.

Reference

1. Roca-Feltrer A, Schellenberg JR, Smith L, Carneiro I. A simple method for defining malaria seasonality. *Malar J* 2009, **8**:276.

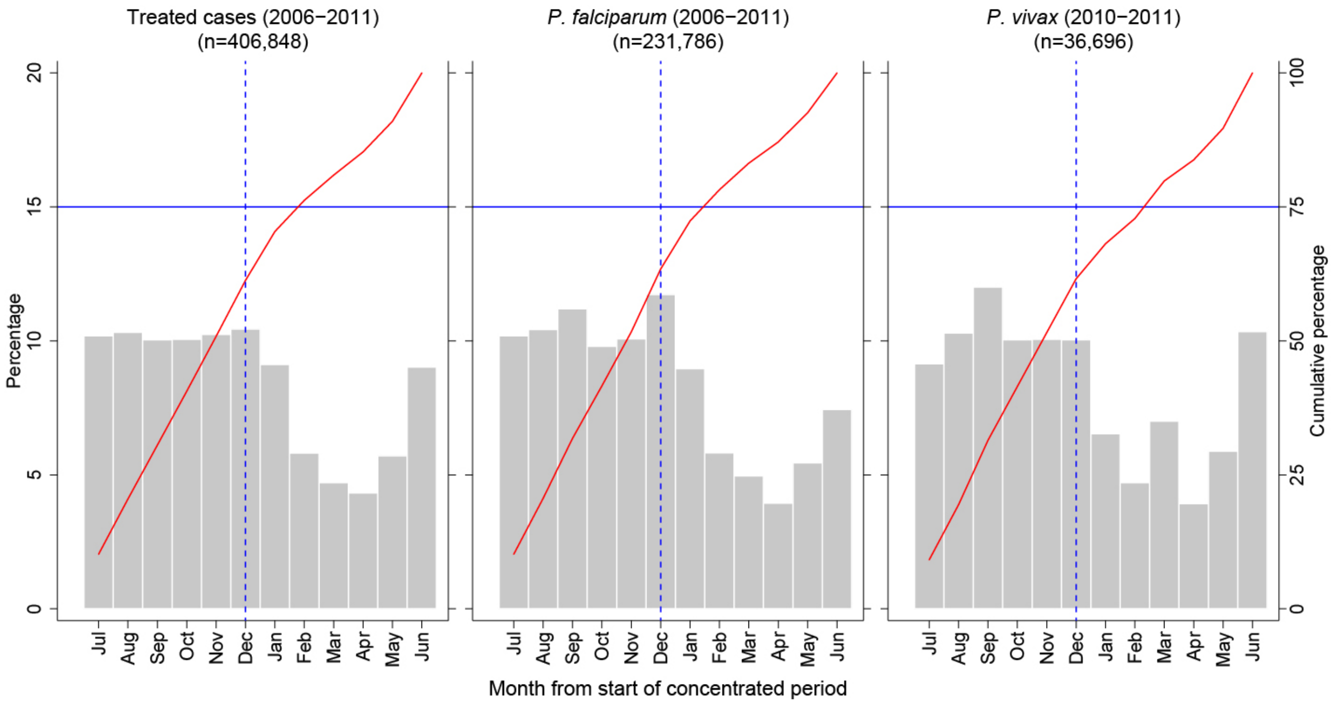

Figure 1. Percentage (left-hand axis) and cumulative percentage (right-hand axis) of treated cases, *P. falciparum* cases and *P. vivax* cases by month from start of concentrated period of cases. The point at which the solid red line crosses the horizontal blue line defines the number of months in which 75% of cases occur. The blue dashed line crosses the horizontal blue line at month 6 of the concentrated period of cases.
