# Supplementary material for: Novel approaches to risk stratification to support malaria elimination: an example from Cambodia
Source: Malar J. 2014 Sep 19;13:371. doi: 10.1186/1475-2875-13-371 (PMC4177243; doi:10.1186/1475-2875-13-371)
Supplement: Supplementary file 2 — Additional file 2: Maps of key district-level outcomes. This file includes maps that illustrate the spatial distribution of three outcomes of the analysis down to the level of the operational district: proportion of villages contributing to the 2011 stratification; malaria incidence in 2011; P. falciparum as a proportion of all malaria cases. (PDF 3 MB) [file 12936_2014_3402_MOESM2_ESM.pdf]

Additional file 2
Maps of key district-level outcomes

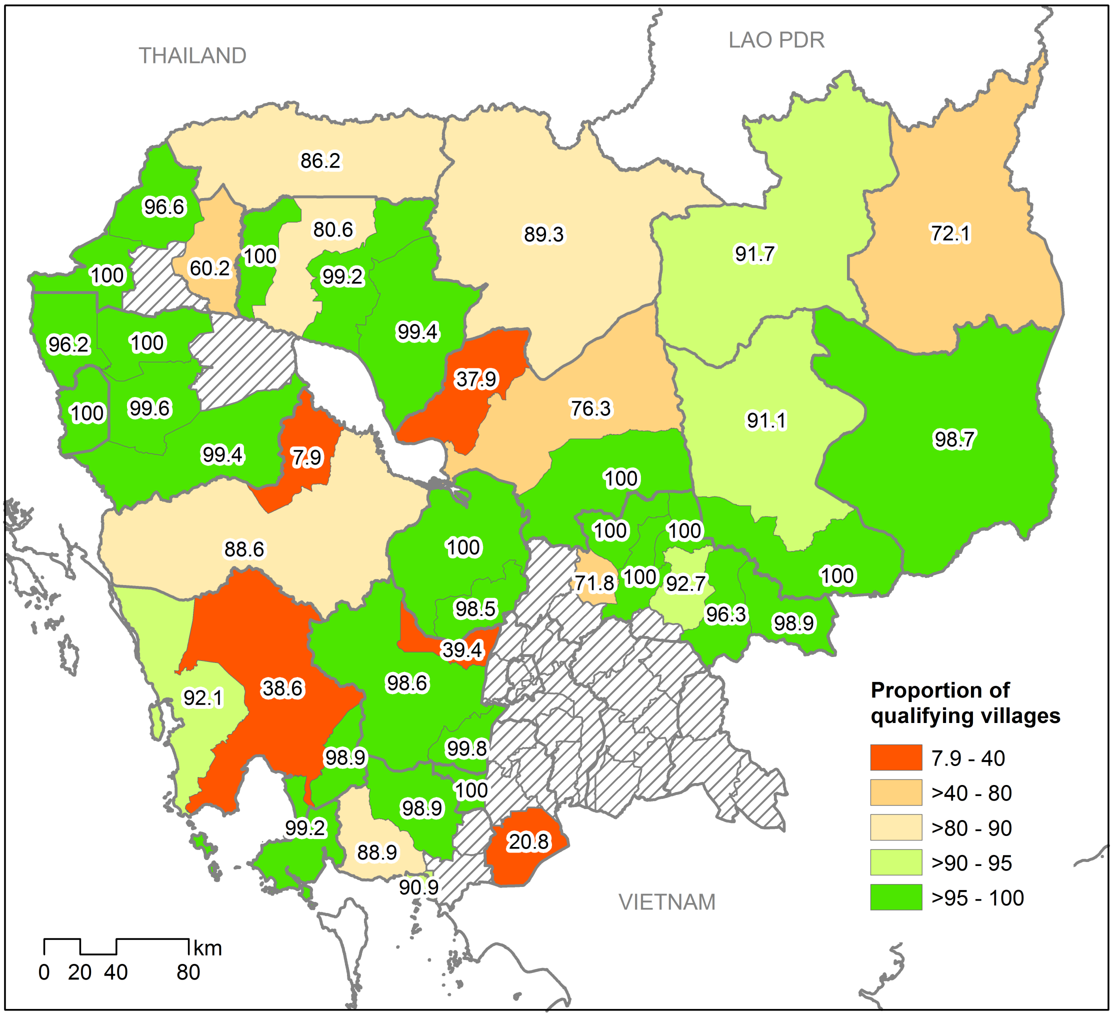

Figure 1. Proportion of villages contributing to the 2011 village-level stratification, mapped by operational district. (Note: districts with grey hatching are not included in the Cambodia Malaria Information System).

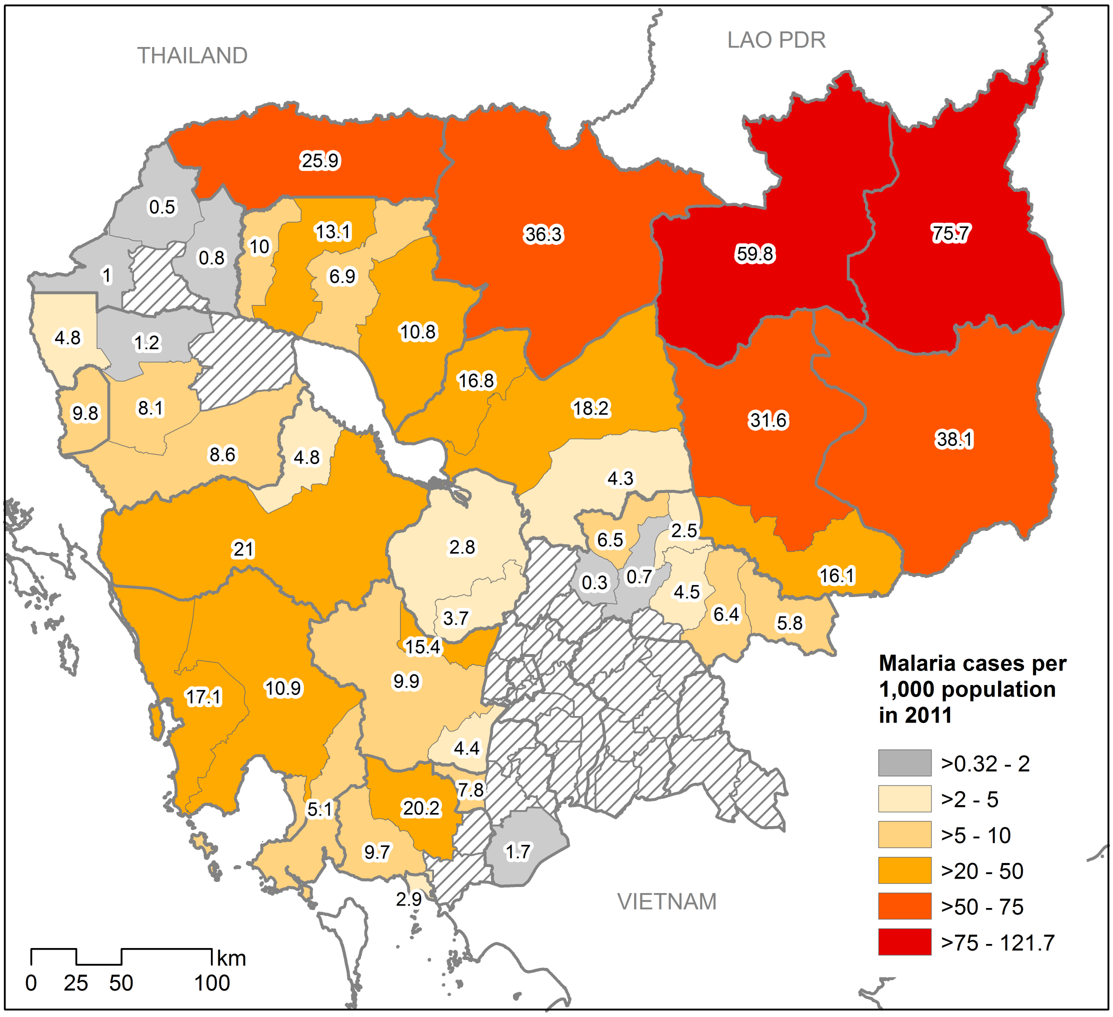

Figure 2. Annual malaria incidence (cases per 1,000 population) for 2011 for all parasite species, mapped by operational district.

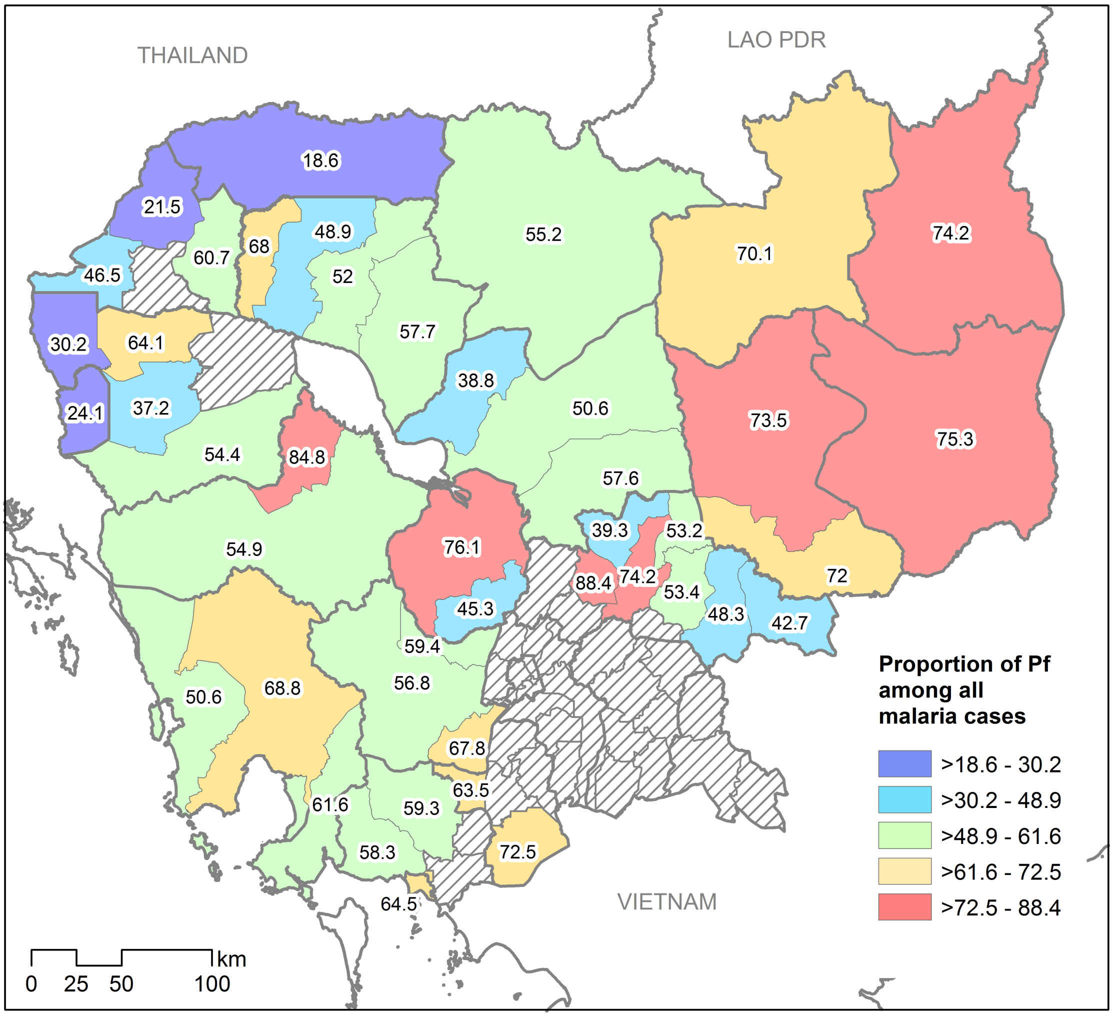

Figure 3. Proportion of *P. falciparum* among all malaria cases reported in 2011, mapped by operational district.
